# Autosomal Dominant Tubulointerstitial Kidney Disease—UMOD: Case Report and Disease Update

**DOI:** 10.3390/diagnostics16101467

**Published:** 2026-05-12

**Authors:** Mario Bonomini, Valeria Vezzani, Michele Rossini, Lorenzo Di Liberato, Liborio Stuppia, Valentina Gatta

**Affiliations:** 1 Department of Medicine and Science of Aging, Nephrology and Dialysis Clinic, SS. Annunziata Hospital of Chieti, “G. D’Annunzio” University of Chieti-Pescara, 66100 Chieti, Italy; valeria.vezzani93@gmail.com (V.V.); lorenzo.diliberato@asl2abruzzo.it (L.D.L.); 2 Nephrology, Dialysis, and Transplantation Unit, Department of Precision and Regenerative Medicine and Ionian Area, University of Bari Aldo Moro, 70121 Bari, Italy; michelerossini@libero.it; 3 Department of Neuroscience, Imaging and Clinical Sciences, “G. D’Annunzio” University of Chieti-Pescara, 66100 Chieti, Italy; stuppia@unich.it (L.S.); v.gatta@unich.it (V.G.); 4 Unit of Molecular Genetics, Center for Advanced Studies and Technology (CAST), “G. D’Annunzio” University of Chieti-Pescara, 66100 Chieti, Italy

**Keywords:** ADTKD-UMOD, autosomal dominant tubulointerstitial kidney disease, chronic kidney disease, uromodulin, genetics, genetic testing, mutation

## Abstract

**Background and Clinical Significance**: Autosomal dominant tubulointerstitial kidney disease caused by a mutation in the uromodulin gene (ADTKD-UMOD) is a rare kidney disorder characterized by progressive tubulointerstitial damage and a slowly progressive loss of renal function. ADTKD is often under-recognized in the clinical setting. Diagnosis of ADTKD-UMOD can be challenging due to its nonspecific symptoms and is confirmed by genetic testing alone. **Case presentation**: We report the case of a 42-year-old male patient referred for evaluation of renal dysfunction, which was accidentally discovered during routine laboratory checks. He had no significant medical history and no known family history of kidney disease or gout. Physical examination was unremarkable. Renal dysfunction was confirmed, with serum creatinine at 1.44 mg/dL and eGFR at 59.5 mL/min/1.73 m^2^. Urinalysis was within physiological limits, proteinuria being 75 mg/day. Uric acid was mildly elevated (7.5 mg/dL) without a history of gout. Other laboratory findings, including autoantibodies, were in the normal range. The patient underwent a kidney biopsy, though it was not diagnostic, showing mild focal tubular atrophy and interstitial fibrosis without glomerular involvement. Immunofluorescence staining was negative for complement and immunoglobulins. Given the above nonspecific findings, the patient was suspected of having possible ADTKD. Genetic investigation using a clinical exome next-generation sequencing approach identified a novel heterozygous missense variant in the UMOD gene (c.409T>C; p.Cysteine137Arginine (p.Cys137Arg)) that is likely pathogenic. The patient is under regular clinical-laboratory monitoring. After one year, his overall health is good, renal function is stable with no proteinuria, and uric acid is mildly increased without gout attacks. **Conclusions**: Increased clinical awareness is crucial for detecting ADTKD-UMOD. Genetic testing can help to resolve clinical diagnostic challenges in patients with unexplained decreased kidney function.

## 1. Introduction

There is increasing evidence to show the important role of genetic testing in diagnosing adults with chronic kidney disease (CKD) of unknown cause, who would otherwise remain without any definitive diagnosis [1]. Genetic analysis is currently the only way to definitively diagnose autosomal dominant tubulointerstitial kidney disease (ADTKD), a heterogeneous group of rare kidney disorders that share the common feature of progressive tubulointerstitial damage [2,3,4]. ADTKD is characterized by slowly progressive loss of renal function, normal- or small-sized kidneys, bland urinalysis, and tubular damage with interstitial fibrosis in renal histopathology, with no significant specific findings [2]. Hyperuricemia, often accompanied by gout, is frequent and usually precedes CKD, which leads to kidney failure in adulthood [4].

The classification of ADTKD, due to the overlap in phenotype characteristics, has been revised into a classification system based on the underlying genetic defect; it also encompasses conditions formerly known as medullary cystic kidney disease and familial juvenile hyperuricemic nephropathy [2]. Genes with disease-causing variants that account for most, though not all, cases of ADTKD include uromodulin (UMOD), renin (REN), hepatocyte nuclear factor 1b (HNF1B), mucin-1 (MUC1) and, most recently, SEC61A1 and DNAJB11 [5]. Other autosomal dominant conditions associated with ADTKD include Alagille syndrome [6], Townes–Brocks syndrome [7], and HDR syndrome [8].

The prevalent causative gene behind ADTKD has been identified as the gene encoding uromodulin, also known as Tamm–Horsfall protein [3,4,9]. Uromodulin, the most abundant protein in normal urine, is a kidney-specific glycoprotein with pleiotropic roles in physiology and pathology, which may play a role in several kidney disorders, including acute kidney injury, CKD and ADTKD [10]. A list of UMOD pathogenic mutations obtained from literature, collaborators, and referral families has recently been reported [11]. Most UMOD mutations are missense variants, clustered in exons 3 and 4, with more than half resulting in the addition or loss of a cysteine residue, leading to misfolding of the uromodulin protein [4,5,11].

We here report a case of ADTKD-UMOD with a novel heterozygous missense variant (c.409T>C: p.Cys137Arg) in the UMOD gene. The nucleotide change T>C at position 409 of exon 3 had not been previously identified.

## 2. Detailed Case Description

A 42-year-old male patient was referred to the nephrology clinic for a recent accidental finding of abnormal renal function. During annual medical and laboratory checkups, a reduction in renal function was found (serum creatinine 1.6 mg/dL, eGFR 52.7 mL/min/1.73 m^2^), associated with mild hyperuricemia (8 mg/dL); the urinalysis was unremarkable. The patient was asymptomatic, and no abnormalities had been detected in previous laboratory investigations, including urinary tests. Impairment of renal function was confirmed one month later, with serum creatinine values of 1.67 mg/dL and eGFR 50.1 mL/min/1.73 m^2^; hyperuricemia was also present (uric acid 8.3 mg/dL), while 24 h proteinuria was 150 mg. Blood electrolytes were within the normal range. A renal ultrasound showed kidneys of normal size with normal thickness and echogenicity of the parenchyma, a right upper polar parenchymal cyst, and no anatomical abnormalities in the urinary system. The patient was then referred to our hospital.

On admission, physical examination revealed blood pressure of 115/75 mmHg, with no signs of lower leg edema or abnormalities in the heart, lungs, or abdomen. He had no significant medical history, including possible gout episodes. A thorough medication history revealed that the patient was taking none. His father suffered from type 2 diabetes mellitus, while his brother suffered from arterial hypertension. There was no known family history of kidney disease or gout. Serum levels of creatinine (1.44 mg/dL, with eGFR 59.5 mL/min/1.73 m^2^) and uric acid (7.5 mg/dL) were slightly elevated. Serum cystatin C level was 1.11 mg/L. Chemical-physical analysis of urine showed no proteinuria or hematuria. Urine sediment analysis was within physiological limits, and 24 h urine protein was 75 mg/day. Arterial blood gas analysis proved unremarkable. Serum complement (C3, C4) and immunoglobulin (Ig) levels (IgG, IgA, IgM) were within the normal range. The search for antinuclear antibodies, anti-DNA antibodies, ENA, and anti-neutrophil cytoplasmic antibodies was negative. No monoclonal bands were disclosed by electrophoresis of serum proteins. The main laboratory findings are shown in Table 1.

**Figure 1:**
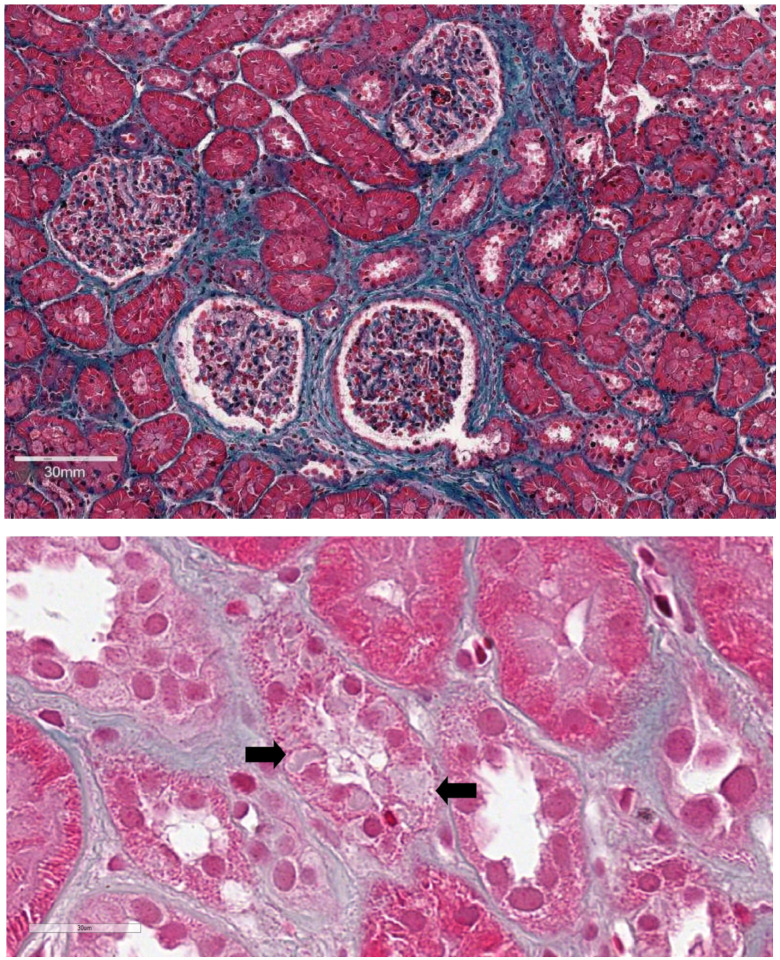
Renal biopsy findings. (**Top**) Low magnification of a renal biopsy showing mild interstitial fibrosis and tubular atrophy along with focal fibrous thickening of Bowman’s capsule without any other glomerular change (Masson’s trichrome). (**Bottom**) Hyalin inclusions (black arrows) in the cytoplasm of tubular epithelial cells of a thick ascending limb of Henle’s loop (Masson’s trichrome).

To identify the etiology and severity of renal involvement, after obtaining informed consent, a renal biopsy was performed in the absence of complications. Light microscopic examination of the kidney biopsy specimen revealed 12 glomeruli per section of cortical parenchyma, one of which was globally sclerotic. The remaining glomeruli showed focal thickening of Bowman’s capsule in the absence of further morphological alterations. Additional findings included mild focal tubular atrophy and interstitial fibrosis (<10%) (Figure 1, top). In just a few tubular profiles of the thick ascending limb of Henle’s loop, hyalin inclusions in the cytoplasm of tubular epithelial cells were noticed on Masson’s trichrome staining (Figure 1, bottom). No significant vascular abnormalities were observed. Immunofluorescence staining was negative for C3, C4, C1q, IgG, IgA, IgM, fibrinogen, and κ and λ light chains.

**Figure 2:**
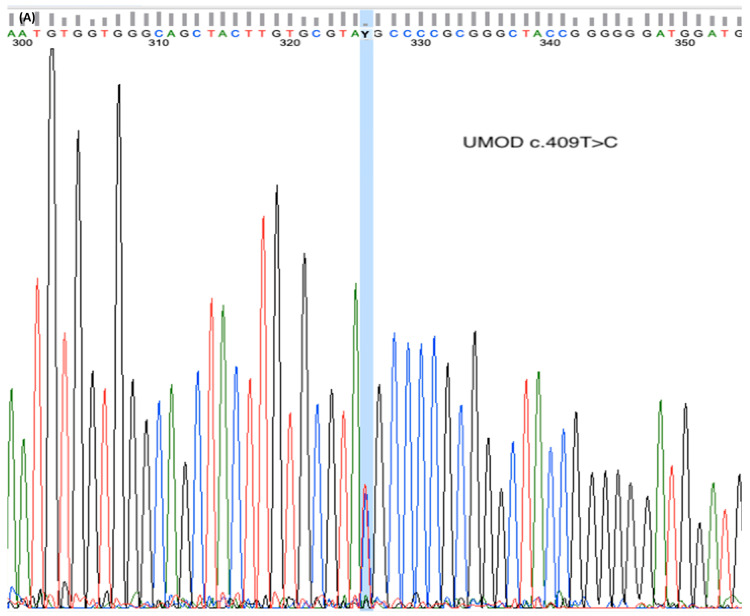

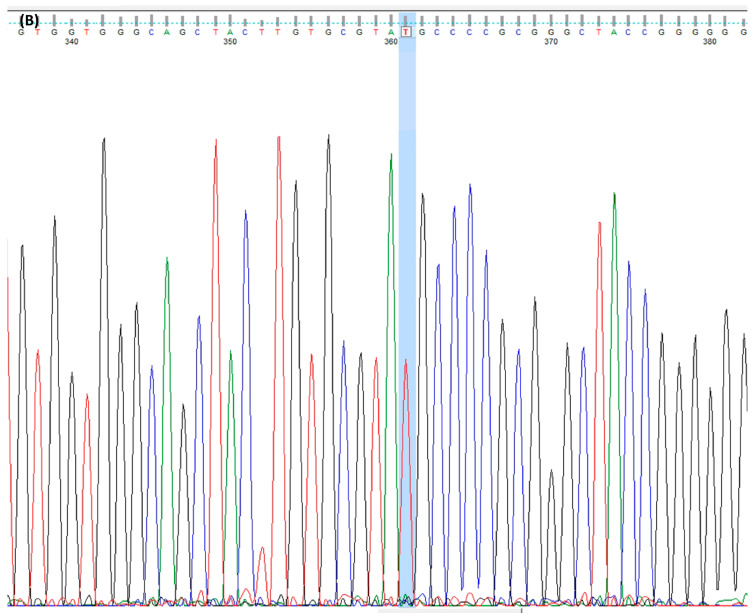
Identification of the UMOD c.409T>C variant by Sanger sequencing. (**A**) Chromatogram of the patient showing the heterozygous T>C substitution in the UMOD gene. (**B**) Sister wild-type chromatogram showing the reference nucleotide T at the same position.

The above nonspecific findings raised a suspicion of ADTKD. A blood sample was accordingly taken for genetic investigation, following the patient’s informed consent. Clinical exome sequencing using a next-generation sequencing (NGS) approach identified a heterozygous UMOD variant (c.409T>C; p.Cys137Arg) (Figure 2A). The result was confirmed by PCR amplification followed by direct Sanger sequencing of exon 3 of the UMOD gene. The analysis achieved a mean coverage of 100× for 98.2% of the targeted regions, and a variant interpretation was performed using Geneyx Analysis (CE-IVD) software (version 6.1). This variant has not been previously reported in the literature or in publicly available databases, including ClinVar and LOVD. According to ACMG/AMP variant interpretation guidelines, supported by in-silico predictions from Franklin Genoox and VarSome, the variant was classified as likely pathogenic. The patient was offered genetic counseling to discuss personal, family, and reproductive risks. Genetic testing of the patient’s parents could not be performed because they were deceased. Segregation analysis in the patient’s sister did not reveal the presence of the c.409T>C variant (Figure 2B), nor did she show any clinical signs or symptoms suggestive of ADTKD-UMOD. The patient has no offspring. Since ADTKD-UMOD has an autosomal dominant inheritance pattern, both the patient and his wife were counseled about the 50% risk of transmitting the gene mutation to their offspring.

At discharge, no drug therapy was prescribed after discussion with the patient and his wife. Inhibitors of the renin–angiotensin system were not started, given the blood pressure values and the absence of proteinuria. The patient preferred not to begin uric acid-lowering therapy. A regular clinical and biochemical follow-up was established.

One year after the diagnosis, the patient had maintained overall good health. Renal function proved stable, with serum creatinine at 1.5 mg/dL and eGFR at 51 mL/min/1.73 m^2^. Uric acid was slightly increased (7.6 mg/dL), with no gout attack occurring. Urinary tests did not show significant abnormalities. Anemia did not occur, with hemoglobin levels at 14 g/dL. Blood pressure remained around 120/75 mmHg.

## 3. Discussion

ADTKD-UMOD is defined by the presence of a heterozygous pathogenic variant in the UMOD gene, which encodes uromodulin [2]. Uromodulin is expressed in the cells of the thick ascending limbs of the Henle loop and early distal convoluted tubules and is secreted bidirectionally into the urine and circulation. Uromodulin is a multifunctional protein, and its function depends on its site of action and form. In the urine, uromodulin maintains homeostasis in the urinary space, protecting against urinary infections and kidney stones. Various protective roles of uromodulin in the interstitium, circulation, and intracellularly have been reported [10]. In ADTKD-UMOD, most experimental evidence suggests that the action mechanism of UMOD pathogenic variants is a gain-of-toxic function [12], with intracellular accumulation of mutant uromodulin [13,14,15,16] inducing endoplasmic reticulum stress and the unfolded protein response pathway, leading to interstitial fibrosis [10]. Recent observations suggest other pathogenic mechanisms, such as complement system activation [17].

The case reported here represents, in our opinion, a frequent challenge to the practicing nephrologist: an adult patient with unexplained decreased kidney function. Despite the absence of a positive family history for renal disorders or gout, ADTKD was considered due to the occurrence of characteristic nonspecific clinical and laboratory findings (decline in renal function, absence of proteinuria, bland urinary sediment, normal-sized kidneys on ultrasound, no history of arterial hypertension, and no exposure to medications potentially causing tubulointerstitial nephritis) as well as histological findings (interstitial fibrosis, tubular atrophy, negative immunoglobulin/complement staining), in the absence of evidence for alternative diagnoses [2]. In this context, it is important to consider other genetic conditions that may affect the differential diagnosis of ADTKD and CKD of unknown origin. Among these, ADTKD-MUC1 represents one of the most relevant alternatives, as it is clinically indistinguishable from ADTKD-UMOD, with slowly progressive CKD and bland urinary sediment. However, molecular diagnosis is challenging due to the VNTR region of MUC1, which is not reliably detected by standard sequencing approaches [18]. ADTKD-REN usually presents earlier in life and is associated with anemia, mild hypotension, hyperuricemia, and low plasma renin levels, which may help distinguish it from UMOD-related disease [19]. ADTKD-HNF1B is typically characterized by a broader and often syndromic phenotype, including renal cysts, congenital anomalies of the kidney and urinary tract, diabetes (MODY5), hypomagnesemia, and genital tract malformations [20]. In addition, multisystem syndromes such as Alagille syndrome, Townes–Brocks syndrome, and HDR syndrome may include renal involvement but are usually distinguishable by the presence of characteristic extrarenal manifestations [6,7,8]. Overall, although these conditions may share overlapping renal phenotypes, careful clinical evaluation and appropriate genetic testing are essential for achieving an accurate diagnosis [21].

Demonstration of a mutation in the UMOD gene led to the diagnosis of ADTKD-UMOD in our patient. Comprehensive genetic testing disclosed the heterozygous missense variant c.409T>C: p.Cys137Arg. The nucleotide change T>C at position 409 of exon 3 represents a novel observation. Segregation analysis showed that the patient’s sister does not carry the variant and does not present clinical manifestations suggestive of ADTKD-UMOD. Although a missed diagnosis in other individuals within the family cannot be unequivocally ruled out (genetic testing could not be performed as the patient’s parents were dead), the genetic defect in the case reported here might represent a *de novo* mutation [22].

The c.409T>C (p.Cys137Arg) variant affects a conserved cysteine residue in uromodulin. Cysteine residues are essential for the formation of intramolecular disulfide bonds as required for correct protein folding, and variants affecting these residues are a well-established pathogenic mechanism in ADTKD-UMOD, leading to misfolding and intracellular retention of mutant uromodulin. The variant is absent from population databases and predicted to be deleterious by in silico tools. Together with the compatible clinical phenotype, this supports classification as likely pathogenic according to ACMG criteria (PM1, PM2, PP3, PP4). However, the pathogenic role of the c.409T>C (p.Cys137Arg) variant should be interpreted with caution. Although classified as likely pathogenic according to ACMG criteria and supported by in silico predictions, segregation data are limited, and no functional studies are currently available. Therefore, a definitive causal relationship cannot be conclusively established, and further studies are needed.

Demonstration of a mutation in the UMOD gene led to our establishing a diagnosis of ADTKD. Such an amino acid change (replacement of cysteine with arginine) without identification of the nucleotide change has previously been reported in a single case, reaching ESRD at the age of 48 years, which is close to the mean age (45 years) of kidney failure in ADTKD [11]. Renal dysfunction, however, was milder in the case presented here and stable over time. Of note, kidney disease progression in ADTKD shows high variability, not only between but also within families, which hampers any accurate establishment of a genotype-phenotype correlation [5].

Our patient is under regular clinical and laboratory monitoring. During a 1-year follow-up, renal function (assessed by eGFR) and uric acid levels (mild hyperuricemia) proved to be stable, with persistent absence of proteinuria. The patient is in good clinical condition, blood pressure remains within the normal range, and no gout attack has developed. Altogether, these findings did not prompt the initiation of any treatment, and this was discussed with the patient.

No disease-specific therapeutic options are currently available for ADTKD-UMOD, and management primarily focuses on slowing CKD progression and treating complications [9]. The main treatment strategies for individuals suffering from ADTKD-UMOD are based on established guidelines for CKD [23]. However, the approach to managing ADTKD-UMOD varies compared to other renal diseases. Diuretics should be used at a minimal dose, as they may aggravate volume depletion and hyperuricemia. Likewise, a low-sodium diet, a common prescription in CKD, is not recommended as it may aggravate hyperuricemia [2]. It is also advisable to avoid adding medications that may worsen interstitial renal disease.

Two recent investigations reported the effects in patients with ADTKD-UMOD of sodium-glucose cotransporter-2 inhibitors, a foundational therapy for CKD. A substudy of the EMPA-KIDNEY trial (Study of Heart and Kidney Protection with Empagliflozin) showed that empagliflozin, compared to placebo, induced a marked reduction (−63%) in urinary UMOD [24]. Note that the two groups were balanced, including genotyping data for UMOD variants that are associated with the excretion of urinary UMOD. Lowering urinary UMOD accounted for 19% of the beneficial treatment effect on the chronic slope of eGFR, comparable to the effect of reductions (15%) in albuminuria [24]. By contrast, a prospective study in ADTKD-UMOD patients treated with empagliflozin or dapagliflozin for more than 1 year showed no improvement in the decline of renal function, though admittedly the study was underpowered [25].

From a genetic counseling perspective, ADTKD-UMOD is inherited in an autosomal dominant manner, implying a 50% risk of transmission to offspring. Therefore, once a molecular diagnosis is established, genetic counseling is strongly recommended to inform patients about disease inheritance, variability in clinical expression, and the potential risk to family members. Cascade testing may be considered in at-risk relatives, even in the absence of overt clinical manifestations. Regarding reproductive aspects, fertility is not typically impaired in individuals with ADTKD-UMOD. However, awareness of the genetic nature of the disease may influence reproductive decisions. In selected cases, options such as prenatal diagnosis or pre-implantation genetic testing for monogenic disorders may be discussed within the framework of specialized genetic counseling. However, caution is warranted when the molecular diagnosis is based on a likely pathogenic variant without any functional validation.

## 4. Conclusions

The estimated prevalence of CKD of unknown origin is 10% to 20%, with up to 20% attributable to a genetic cause [26]. In the absence of evidence for kidney disease of any other etiology, and even without a positive family history, ADTKD should be considered in single cases (like our patient) presenting nonspecific clinical and histological findings [2]. ADTKD is being increasingly recognized given the enhanced availability of genetic testing and better understanding of this condition, and accounts for approximately 5% of all monogenic kidney disorders [3]. The present report shows that genetic testing can help to resolve clinical diagnostic challenges in unexplained CKD. Although ADTKD lacks any effective specific treatment, the reasons for pursuing definitive diagnosis by genetic testing may include confirmation of the diagnosis in the affected patient, information on disease prognosis and clinical management, and indication for genetic counseling for family members. Genetic testing may also direct patients to suitable clinical trials and targeted therapies [21]. The landscape of ADTKD-UMOD research is evolving [9], hopefully leading to clinical approaches that are effective in managing the disease.

## Figures and Tables

**Table 1:** Laboratory data.

Item	Value	Normal Range
Creatinine	1.34 mg/dL	0.6–1.3 mg/dL
Urea	40 mg/dL	17.1–49.2 mg/dL
Cystatin C	1.11 mg/L	0.65–0.9 mg/L
Uric acid	7.5 mg/dL	3.5–7.2 mg/dL
Glycemia	80 mg/dL	70–100 mg/dL
Hemoglobin	14.5 g/dL	13–17 g/dL
Total protein	7.4 g/dL	6.4–8.3 g/dL
Albumin	4.8 g/dL	3.5–5.2 g/dL
Total cholesterol	222 mg/dL	0–200 mg/dL
LDL cholesterol	159 mg/dL	0–100 mg/dL
HDL cholesterol	46 mg/dL	40–60 mg/dL
Triglycerides	141 mg/dL	0–150 mg/dL
Sodium	140 mmol/L	136–145 mmol/L
Potassium	3.9 mmol/L	3.5–5.1 mmol/L
Calcium	9.2 mg/dL	8.4–10.2 mg/dL
Magnesium	1.71 mg/dL	1.6–2.6 mg/dL
Platelets	198 × 10^3^/mmc	150–450 × 10^3^/mmc
White blood cells	8010/μL	4000–10,000/μL
HS C-reactive protein	0.59 mg/L	0–5 mg/L
Lactate dehydrogenase	170 IU/L	125–220 IU/L
Fibrinogen	243 mg/dL	180–400 mg/dL
Complement factor C3	99 mg/dL	82–113 mg/dL
Complement factor C4	20 mg/dL	15–57 mg/dL
Urinary β2MG	0.15 mg/L	0.10.–0.32 mg/L

HS, high sensitivity; β2MG, beta-2 microglobulin.

## Data Availability

The data presented in this study is available on request from the corresponding author.

## Abbreviations

The following abbreviations are used in this manuscript:

ADTKD	Adult Dominant Tubulointerstitial Kidney Disease
CKD	Chronic Kidney Disease
eGFR	Estimated Glomerular Filtration Rate
UMOD	Uromodulin
